# An Update on Planned Oocyte Cryopreservation (POC) in Italy: Medical, Epidemiological and Legal Consideration

**DOI:** 10.3390/ijerph19042371

**Published:** 2022-02-18

**Authors:** Jessica Cremonese, Marianna Marcon, Laura Oppi, Giulia Paletti, Vincenzo Romolo, Pamela Tozzo, Luciana Caenazzo

**Affiliations:** 1 Galileian School of Higher Education, University of Padova, 35121 Padova, Italy; jessica.cremonese@studenti.unipd.it (J.C.); marianna.marcon.1@studenti.unipd.it (M.M.); laura.oppi@studenti.unipd.it (L.O.); giulia.paletti@studenti.unipd.it (G.P.); vincenzo.romolo@studenti.unipd.it (V.R.); 2 Legal Medicine Unit, Department of Cardiac, Thoracic, Vascular Sciences and Public Health, University of Padova, 35121 Padova, Italy; luciana.caenazzo@unipd.it

**Keywords:** women health, Planned Oocyte Cryopreservation, oocyte biobanking, Italy, ethics

## Abstract

Starting with a brief socioeconomic analysis of the phenomenon of female fertility, this narrative review aims to provide an analysis of the use and possibilities of medically assisted reproductive technology in combating fertility issues, adopting socioeconomic, legal and medical perspectives in Italy. The authors mainly employ data from the annual reports of the National Registry of Medically Assisted Reproduction (PMA Registry) and the Italian Statistical Institute (ISTAT) to understand the evolution of oocyte use in medically assisted reproductive technology in Italy from 2015 to 2018 and in particular to dissect the possibilities of oocyte cryopreservation as a measure to counteract age-related infertility, specifically through Planned Oocyte Cryopreservation (POC), also known as “social freezing”. It seems that the best course of action in the context of medically assisted reproduction would be the use of young and healthy cryopreserved oocytes (autologous or donated), preferably before the age of 40, while encouraging donation of oocytes whenever possible. Italy’s dependence on foreign biobanks for donated oocytes calls for the institution of a national biobank and further specific regulation of gamete donation. For this reason, it would be useful to encourage the acceptance of Planned Oocyte Cryopreservation to allow greater availability of healthy, younger oocytes.

## 1. Introduction

The decline of birth rates is an issue which has long involved social scientists and the medical community. Fertility rates are impacted by a number of variables, among which the most frequently observed are maternal human capital, participation in the work force, surrounding institutions and social norms. The fertility level of countries such as Italy has been persistently below 1.5 children per woman for over 20 years, a level considered to be “very low” and decidedly below the generational turnover threshold of 2 [1].

A clear difference emerges between European countries which exhibit a fertility rate above the replacement level and those who do not, with Italy at the bottom of the list. In Italy the fertility rate has remained decidedly below 1.5, and the average maternal age has steadily risen over the last 20 years [1]. The medical literature extensively shows how maternal age is strongly associated with pregnancy and birth complications such as prematurity, difficult labor and lower childbirth weight [2]. Furthermore, a higher age at the first birth also impacts the number of children per woman since fertility declines significantly with age and less time remains to have multiple children, even if we do not consider individual preferences as to the quantity of offspring.

This postponement of maternity was first defined as a “postponement transition” by Kohler et al. when describing the tendency to move family formation from the younger to the older years of a woman’s life [3]. A delay in childbearing may arise from a number of interacting factors. Besides the obvious direct costs of pregnancy, we can point to the competition of parenthood with educational and career aspirations, financial insecurity, value changes, housing conditions and a lack of institutional support. In the Italian case, proof of fertility postponement emerges from the rising maternal age.

Gustafsson indicates the increase in work force participation and higher average educational achievement as causes of this phenomenon, with more highly educated women having fewer children later than their less-educated counterparts [4]. The role of education in fertility is well-documented, with a negative correlation between educational maternal scholastic achievement and the birth rate, as well as higher ages when bearing a first child than more educated cohorts both in developed and developing countries [5]. In turn, better-educated women seem to obtain better jobs and to accumulate more skills and experience than their counterparts with children [6]. Caltabiano et al. are among the very few authors indicating a possible inversion in the above-mentioned trend, stressing a North–South divide in the fertility trend on the Italian peninsula [7]. In recounting the history of fertility in the two areas of the country, the authors set out differences in cohort fertility rates dating back to the thirties. The difference in trends extends to more recent times, when the authors noticed a recovery in fertility in the northern part of the country, as opposed to a continuing decline in the southern regions’ fertility. Still, their conclusion stresses the postponement tendency.

The role of institutions and social norms as determinants of childbearing’s explicit and implicit costs has been discussed by Adserà. The author found that OECD countries with low unemployment and institutions that favor a flexible job market tend to have fertility rates around the replacement rate, in contrast with countries where the implicit and explicit costs of pregnancy and parenthood are worsened by unemployment and a rigid job market [8]. In Italy, support for families is almost entirely at the expense of families who have to compensate themselves for the lack of social services. Recent studies have shown that policies that offer affordable childcare opportunities are one of the main incentives for the participation of mothers in the world of work. In particular, while men’s employment rate is hardly affected by childcare costs, the mother’s chances of full-time work significantly increase if adequate financial support and childcare assistance is provided [9,10,11].

The “second demographic transition” theory attributes falling fertility rates from the 1960s to a shift towards a more individualistic family model. This entails greater desire for self-realization, emancipation and personal development, which in turn affects when and with whom women may decide to build a family. With time, the preference has moved towards a smaller family structure, sometimes with preferences for a below-replacement number of children [12]. Testa reported answers to the Eurobarometer survey of 2006 to questions such as “In your opinion, what is the ideal age for a woman to have her first child?”, to which most Europeans responded with a desire to start a family later in life, which is consistently reflected in the aforementioned data [13].

The advent and diffusion of Assisted Reproductive Technologies (ART) may have led to a stronger belief that parenthood postponement lessens the biological limits of human reproduction. A lively debate has been triggered on so-called “social egg freezing”, or on the possibility of the use of the cryopreservation technique by healthy and currently fertile women, who wish to postpone pregnancy for nonmedical but social reasons. The choice to postpone motherhood can result from a variety of factors, such as the lack of a suitable partner, unstable financial situations, housing issues and conflicts with educational and career aspirations [14,15]. Looking specifically at assisted reproductive technologies, the possibility of cryopreserving oocytes with the aim of future fertilization and pregnancy today appears preferable for many women rather than the possibility of freezing embryos with a view towards preserving future fertility.

## 2. Methods

In the paragraphs below, our analysis will first focus on the Italian legal perspective on Planned Oocyte Cryopreservation (POC). Secondly, we will focus on the medical perspective and on POC as a medical treatment for age-related fertility preservation, with a focus on the efficiency and safety of POC. Finally, the analysis of the ethical implications of this issue will start with a synthesis of the positions adopted by the Italian Committee for Bioethics (CNB) discussing some ethical implications of considering POC as a preventative measure of infertility. Data analysis will be provided starting with the Italian Assisted Reproductive Technology Register (IARTR) Executive Summary for 2018 [16], which provides regular executive summaries with data on the number of active centers. IARTR belongs to the Italian National Institute of Health (ISS), and the last available published data of IARTR executive summaries are those from 2018.

## 3. Legal Approach to Planned Oocyte Cryopreservation in Italy

From a legal perspective, the phenomenon of POC has not yet received much attention in Italy, hence the necessity of examining in which ways this practice is in line with current legislation. It is of the utmost importance to understand whether POC for nonmedical reasons falls under the scope of Italian Law no. 40, adopted on 19th February 2004, entitled “Rules on medically assisted procreation” [17]. Any conception resulting from a medical treatment and not from sexual intercourse can only qualify as ART. This opinion has been shared by the Italian Council of State, the highest administrative judicial body in Italy [18,19]. Accordingly, as far as Law 40/2004 is concerned, access to ART techniques is an option only if it follows a documented or certified diagnosis of infertility or sterility, as set out in Article 4, or if the couple is fertile but affected by transmissible genetic diseases [20]. Moreover, this law enshrines the principle of gradualness, meaning that priority should always be given to less invasive and less complex procedures. In particular, the guidelines of the Ministry of Health provide three levels of ART techniques; it should be noted that the two techniques involved in social freezing, namely oocyte collection and embryo transfer, are considered to be Level II and Level III techniques, respectively [21].

Consequently, the future use of oocytes that have been previously cryopreserved for nonmedical reasons is allowed only under strict conditions. Firstly, there must be a condition of supervening sterility or infertility, seen as an inability to conceive after at least one year of unprotected sexual intercourse, as explained by the guidelines. Secondly, respect for the principle of gradualness suggests that other less invasive procedures should be attempted prior to the transfer of the cryopreserved oocytes; those techniques include, for example, intrauterine or intraperitoneal insemination [21]. The regulatory background on POC in Europe is highly heterogeneous and varies from strict legislation forbidding this practice to the absence of specific regulation in 12 countries. Indeed, nonmedical oocyte freezing is not permitted in Austria, France, Hungary, Lithuania, Malta, Norway, Serbia or Slovenia, and it is not performed in Bosnia–Herzegovina and Moldova, even in spite of the absence of specific legislation [22,23].

From a constitutional standpoint, it is well-known that the right to self-determination and the right to health are fundamental rights in Italy. On the one hand, self-determination is meant, in its family dimension, as a right to be a parent, and it has its constitutional basis in Articles 2, 3 and 31 of the Italian Constitution [24], as well as in Articles 8 and 14 of the European Convention of Human Rights [25,26]. On the other hand, the right to health, as set out in Article 32 of the Italian Constitution, cannot only be intended as a right to physical wellbeing. The Italian Constitutional Court has established that this particular right shall be read as including mental health [24,27,28,29], whose protection must be equal to that of physical health, given that the impossibility of having children through ART can have a relevant negative effect on couples’ health [30]. The content of this right should be read in light of what science, meaning the medical–scientific shared literature, considers good or bad for health. Scientific evidence becomes an interposed parameter of constitutionality that the legislator is urged to respect [31]. Thus, an intervention regarding the appropriateness of therapeutic choices should not depend on mere political discretion but should be the result of guidelines based on the state of scientific knowledge, as gathered through national and international institutions and organizations in the field [32].

Given the necessity of the legitimacy of POC [33], introducing legislation for regulating this practice would not only be compatible with the Italian Constitution, but it would also make constitutional rights effective. This seems to be the logical consequence of what has been stated by the Constitutional Court in Decision no. 162/2014, whereby the choice to be parents and to start a family with children has to be regarded as an expression of the general fundamental freedom to self-determination, referring to Articles 2, 3 and 31 of the Italian Constitution, since it concerns the private family sphere [24]. Thus, the decision whether to have a child or not cannot be coercible as long as it does not affect other constitutional rights.

In addition, the overwhelming majority of donated oocytes in Italy, up to 93% over the 2015–2018 period, came from foreign biobanks [34]. Given the necessity of oocyte donation for women undergoing IVF at an advanced age, a specific regulation of this practice and of oocyte biobanks would open the way to an increased number of cryopreserved oocytes available for donation in our country. These regulatory interventions could also be accompanied by incentive campaigns aimed at encouraging donation from women that opted for POC at a young age and that are no longer in need of those previously stored oocytes [35]. For instance, a form of incentive proposed in the literature consists of the offer of so-called “freeze and share” programs, which allow for the donation of part of the cryopreserved oocytes (and, more specifically, those that are no longer needed by the woman) in exchange for discounts in gynecological treatments for the donor [36]. Moreover, these incentives could partially solve the problem related to the increasing demand for donated oocytes and the common reluctance of women to donate the ones that remain unused after POC [37,38]. On the other hand, one can envision that women in need of expensive procedures not covered by medical insurance would opt for “forced” donation to cover medical bills.

Therefore, at the present time, lack of regulation for POC in Italy presumably discourages this practice—as well as the difficulty of coordination with the rules laid down in Law no. 40/2004. This could also be a possible explanation for the distinct lack of cryopreserved oocytes available for donation, whose number is likely to increase with clear POC legislation. A delay in the regulation of bio-law matters and an imposition, when a decision is taken, of a unilateral ethical view from above—which brings about legal approximation and ideological hubris [39]—can only result in assigning to courts and judges the burden of filling legislative vacuums and fixing distortions of the regulation, thus raising relevant issues of institutional balancing between *iurisdictio* and *gubernaculum* [40]. As a consequence, considering the absence of a constitutional impediment and the ever-increasing social demand for the practice of POC, a comprehensive regulatory intervention in this field may be required in order to recognize the practice of POC for nonmedical purposes and to fill the above-mentioned regulatory gap.

## 4. Assisted Reproductive Technology in Italy

In this framework, which has emerged in recent decades, we should factor in the introduction of assisted reproductive technologies, such as in vitro fertilization (IVF), embryo and oocyte cryopreservation and hormonal fertility therapy. These medical interventions aim at offering a solution to fertility issues, whether caused by situations such as supervening illness or by the decrease in fertility determined by general biological characteristics and genetic predisposition to age-related fertility decline. The definition of infertility, as adopted by the 2015 Italian Decree no. 161 containing guidelines on ART procedures, is the absence of conception, aside from cases of recognized pathology, after 12 to 24 months of regular unprotected intercourse in heterosexual couples [21]. The treatment of infertility includes restoring fertility, surgically intervening to restore fertility and access to ART. The main driver of infertility is the age of the woman, with a higher risk of spontaneous termination of pregnancy for women above 30 and a reduction of 50% in the probability of pregnancy in couples with women above 35. Besides age, the impact of congenital follicular heritage could be lower than demographically expected because of genetic predisposition or interference of several factors such as lifestyle, drug therapies and environmental determinants. Among other causes of infertility there figure clinical conditions such as endometriosis, fibromas, tubal diseases, anovulation often derived from diseases such as polycystic ovary syndrome and contracting sexually transmitted diseases. Overall, female infertility is responsible for 35–40% of couples’ infertility.

In Italy, the Italian Assisted Reproductive Technology Register (IARTR) provides regular executive summaries with data on the number of active centers providing data for statistical purposes [16]. In 2018, there were 345 active centers for ART in Italy, of which 202 deal with second- and third-level procedures, which require local anesthesia and/or deep sedation and general anesthesia with intubation such as in vitro fertilization (FIVET), intracytoplasmic sperm injection (ICSI), oocyte retrieval, gamete intrafallopian transfer (GIFT), microsurgical and non-microsurgical testicular gamete withdrawal and laparoscopic gamete withdrawal [21].

**Figure 1:**
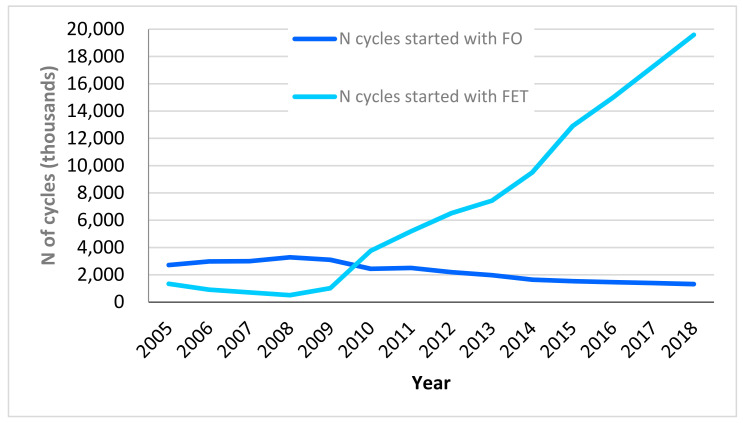
Number of started cycles with frozen oocytes (FO) and frozen embryos (FET), 2005–2018. Italian data extracted from IARTR. The Italian Assisted Reproductive Technology Register. Executive Summary for 2018. Available online at https://www.iss.it/documents/20126/0/EXECUTIVE+SUMMARY+of+ART+in+ITALY+-+Activity+2018_.pdf/06d0b225-ac13-9be0-c20c-c94dfb6e3547?t=1617357530502 (accessed on 28 November 2021).

Using data from the IARTR to observe the employment of different fertility aide techniques in Italy over the last decade, we can analyze changes in trends. Considering data for the 2005–2018 period, as presented in the IARTR Executive Summary for 2018, thawing techniques include frozen-thawed embryo transfer (FET) and frozen oocyte (FO); fresh techniques include in vitro fertilization and embryo transfer (FIVET) and intracytoplasmic sperm injection (ICSI). Among frozen techniques, a key change has been taking place since 2010: FO has been slowly declining in application as opposed to a steep increase in FET employment (Figure 1). Focusing on the donation of oocytes, we know that the overwhelming majority of donated oocytes used in Italian ART procedures are thawed and come from foreign biobanks. In conjunction with the improvement in the efficacy of thawing-based techniques, the foreign sourcing of donated gametes creates inefficiency in the Italian ART panorama since demand for frozen oocytes is increasing, and availability does not respond accordingly. The result is an increasing dependency on foreign biobanks.

## 5. POC as a Medical Treatment for Age-Related Fertility Preservation in Italy

The literature clearly demonstrates that the chances of achieving pregnancy and the birth of a healthy child without complications vary greatly with ageing. Advanced maternal age has been linked to numerous issues, among which is the shortening of offspring lifespan [41].

The reproductive efficiency of females correlates negatively with age because of the exponential decrease in ovarian reserve from the age of 37 and onwards [42].

Furthermore, the development of the embryo can be affected by both intrinsic and extrinsic factors, such as environmental conditions during fetal development, which seem to have an effect on the epigenetic modifications [43]. As for the intrinsic factors, we know that the egg is the unique contributor of mitochondria to the embryo and that these organelles contain DNA sequences that are independent of the genome. According to Wilding et al., since the genetic material contained in the mitochondria degenerates with age, advanced maternal age could, in this perspective, therefore be considered to be a “genetic disease” [41]. It has indeed been demonstrated that a progressive degeneration of the respiratory capacity of mitochondria in the eggs of women of advanced fertile age results in an energy deficit and consequent secondary effects on the oocyte and developing embryo. This can be explained by the way mitochondria’s aerobic respiration works to produce ATP as well as free radicals that may attack proteins and mitochondrial DNA, leaving cumulative damage, leading to mutations and, therefore, a reduced efficiency associated with ageing [44,45,46]. Ageing is also associated with an increase in the frequency of aneuploidy in the oocyte through errors in meiosis [47]. Moreover, the risk of chromosomal disorders such as Down’s syndrome increases with age from 1 in 1500 at age 20 to 1 in 35 at age 45 [48,49].

Taking into consideration all these biological aspects, Planned Oocyte Cryopreservation allows women to overcome this condition and bear a child past the point of their fertility decline, without all the risks connected to delaying pregnancy. Egg freezing for nonmedical reasons can therefore be considered a method of allowing a healthy fertility in a precautionary way, and in this view of the matter we might define it as a medical treatment since it prevents a pathological condition such as infertility. On the other hand, it is fundamental to consider that egg freezing cannot safeguard against all age-related fertility issues. Thus, the real effectiveness of the procedure should be correctly reported to possible users to prevent the encouragement of delayed pregnancies and distorted expectations about risks and efficacy under the promise of greater reproductive autonomy.

### 5.1. POC and ICSI Efficiency and Safety

**Figure 2:**
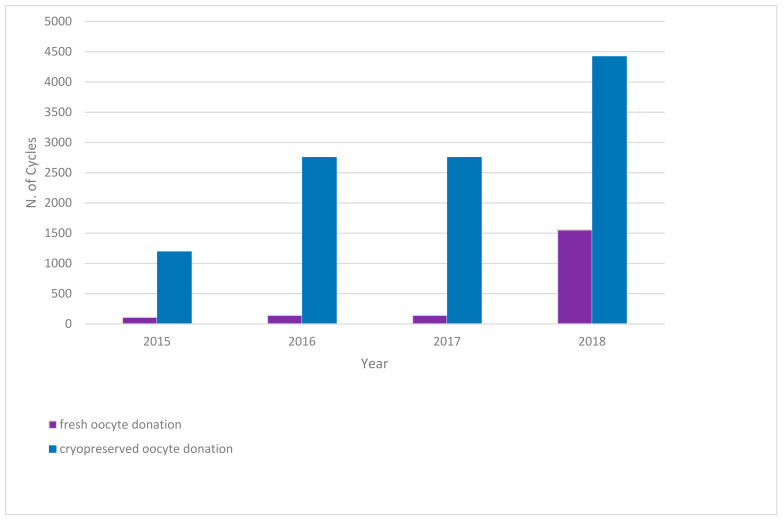
Increase in implementation cycles from fresh and cryopreserved oocytes, 2015–2018. Blue bars represent cryopreserved oocyte donation, purple bars represent fresh oocyte donation. Italian data extracted from IARTR. The Italian Assisted Reproductive Technology Register. Executive Summary for 2018. Available online at https://www.iss.it/documents/20126/0/EXECUTIVE+SUMMARY+of+ART+in+ITALY+-+Activity+2018_.pdf/06d0b225-ac13-9be0-c20c-c94dfb6e3547?t=1617357530502 (accessed on 28 November 2021).

ART techniques have greatly improved in the last few years, with oocyte cryopreservation becoming an important component of it and a consequent increase in women’s demand for POC, allowing them to prevent childlessness due to age-related fertility decline. In Europe, the regulatory framework differs significantly across countries, creating a mosaic of situations. As of 2017, no member states had offered public funding for nonmedical POC. Three member states—Austria, France and Malta—prohibited egg freezing for nonmedical purposes, and most countries refrained from providing indications on the matter of elective nonmedical egg freezing [22]. Nevertheless, oocyte cryopreservation for medical and nonmedical purposes has gained traction inside and outside of Europe as techniques have improved. Some countries have reported extreme increases in egg freezing: for instance, the US alone reported an 880% increase over the period 2010–2016 in the use of egg freezing [50], sometimes with workplaces providing the possibility of undergoing the procedure to their employees as a nudging policy [51]. In Italy, the growing activity surrounding ART is reflected by the numbers of the IARTR Executive Summaries from 2015–2018 (see Figure 2).

Some have speculated that this so-called “social freezing” may be intentionally used by women as insurance against future age-related infertility, allowing them to maintain the possibility of conceiving children even after their physiological fertility decline and possibly encouraging delayed pregnancies [14]. However, it might be argued that this is a false sense of security and that egg freezing is an imperfect solution to the problem of postponing childbearing [15]. Therefore, it should be clear to women who undergo POC in order to have children at a later age with ICSI what the actual associated rates of successful pregnancies are and what the risks are. To understand the real effectiveness of frozen oocyte procedures, we examined percentages of pregnancies over oocyte transfers for the period 2015–2018 using data from the IARTR 2018 Executive Summary [34]. The ratio of pregnancies over oocyte transfers in the reference period was always below 40% and quite stable over time for both fresh oocyte and thawed oocyte procedures. In regard to Planned Oocyte Cryopreservation, a healthy woman of ideal fertile age undergoing the procedure who is aware of this data is not likely to perceive 40% as an assurance of pregnancy, especially within the Italian regulatory framework where the cryopreserved oocytes could be accessible for autologous use only if the patient has the required criteria to access such second- and third-degree procedures.

To further discuss their effectiveness from a technical point of view, we considered that the entire process of POC and ICSI comprises the following steps:

1. Ovarian stimulation and oocyte retrieval;
2. Cryopreservation and storage;
3. Thawing, fertilization of oocytes and implantation.

We can take these steps as endpoints to review the safety and efficacy of the current techniques in Italy.

#### 5.1.1. Ovarian Stimulation and Oocyte Retrieval

The controversy over POC lies in applying a medical treatment to a healthy woman, considering the risks related to the process of ovarian stimulation and oocyte retrieval [52,53], since there are associated risks of drug reactions, surgical complications and ovarian hyper-stimulation syndrome (OHSS). In particular, concerns have been raised around the use of GnRH antagonists, which expose the patient to high levels of estradiol [54], but these can be blocked with the use of aromatase inhibitors in patients who might have estrogen-sensitive disorders, so that ovarian stimulation is overall considered to be a safe procedure.

#### 5.1.2. Cryopreservation and Storage

Concerning cryopreservation, storage and thawing of the oocytes, the frozen oocyte (FO) survival rate in Italy improved from 55.2% in 2005 to 67.2% in 2013 [34], while other literature reports an oocyte survival rate of over 84% [36], and, applying the Cryotop method, 91% [55] and 94% [56], respectively.

Oocytes are difficult to cryopreserve since they have a high susceptibility to intracellular ice formation, which may damage the microtubules that have a fundamental role in normal chromosomal segregation, but a great improvement has been made since vitrification, and the Cryotop method in particular has become the technique of choice for cryopreservation of oocytes instead of slow freezing [57,58,59,60].

#### 5.1.3. Thawing, Fertilization of Oocytes and Implantation

As for the fertilization of oocytes and implantation, the combination of vitrification and ICSI in vitrified oocytes guarantees the best pregnancy outcome [61]. IVF/ICSI clinical pregnancy rates and live birth rates using vitrified oocytes are now comparable to those obtained by fresh cycles [36,62,63]. Fertilization rates are 76.3% and 82.2% for vitrified and fresh oocytes, respectively [64].

Considering embryo transfer, different regulatory frameworks imply a heterogenicity in the number of embryos transferred per cycle. According to a study published in 2020 by the European IVF-monitoring Consortium (EIM) for the European Society of Human Reproduction and Embryology (ESHRE), most transfers involved the replacement of two embryos (51.9% of the transfer cycles), and the proportion of transfers of only one embryo per cycle has been increasing since 2015 (41.5% vs. 37.7% in 2015), and the number of transfers of three or more embryos has continued to decrease. In the same study, Serbia reported more than 50% of transfers with three embryos, while the highest proportion of transfers of four or more embryos was recorded in Greece [65].

**Figure 3:**
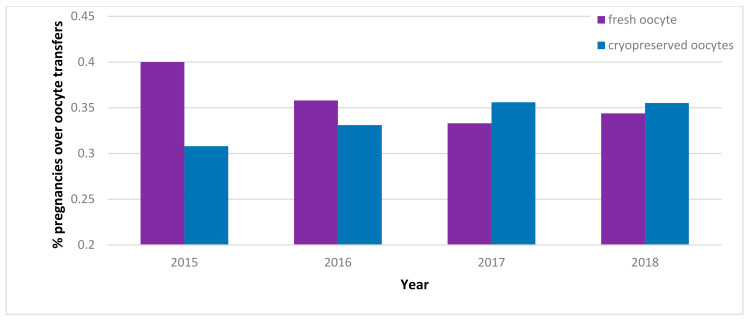
Pregnancies per oocyte transfers, 2015–2018. Italian data extracted from IARTR. The Italian Assisted Reproductive Technology Register. Executive Summary for 2018. Available online at https://www.iss.it/documents/20126/0/EXECUTIVE+SUMMARY+of+ART+in+ITALY+-+Activity+2018_.pdf/06d0b225-ac13-9be0-c20c-c94dfb6e3547?t=1617357530502 (accessed on 28 November 2021).

**Figure 4:**
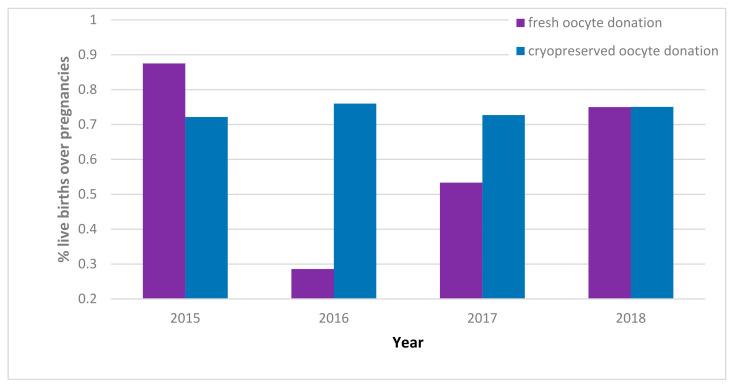
Live births per pregnancy, 2015–2018. Italian data extracted from IARTR. The Italian Assisted Reproductive Technology Register. Executive Summary for 2018. Available online at https://www.iss.it/documents/20126/0/EXECUTIVE+SUMMARY+of+ART+in+ITALY+-+Activity+2018_.pdf/06d0b225-ac13-9be0-c20c-c94dfb6e3547?t=1617357530502 (accessed on 28 November 2021).

Relying on report data from the IARTR for the 2015–2018 period, we can also observe the aforementioned pregnancy rate for embryo transfer (Figure 3) and the number of live births (viable birth at >24 weeks of gestation) over pregnancies to evaluate the efficiency and efficacy of procedures in Italy (Figure 4). The scarcity of fresh oocyte transfers observations for the years 2015–2017, with less than 140 transfers for these three years, contrasts with the jump to 1524 transfers in the year 2018. This is an issue to note when interpreting the ratios presented in our graphs.

While pregnancy over oocyte transfer rate is stable across the years for both fresh and thawed procedures, there is an inconsistency across the ratio of live births over pregnancies for fresh procedures, which may be attributed to the scarcity of observations. Instead, the cryopreserved oocyte live births over pregnancies ratio appears stable over the years and is 74% on average.

Looking at the safety of this technique in terms of newborns’ health, we can consider the follow-up to babies born from cryopreserved oocytes. Data from 2005 to 2013 show that fetal and perinatal complications do not differ between pregnancies from frozen-thawed and fresh oocytes [66].

As for the rate of neonatal major congenital anomalies, it does not differ between children born from oocyte vitrification and children born naturally or using standard IVF [67,68]. In Italy, there is indeed a very low rate of congenital anomalies reported (0.9%) involving oocyte cryopreservation, with no significant increase in the risk of birth defects among births resulting from IVF and ICSI as compared with births to fertile women that did not involve ART [34] (Table 1).

Moreover, a number of studies have registered obstetric and perinatal outcomes of babies born from vitrified oocytes, comparing them to those achieved with fresh oocytes.

No differences were found in terms of obstetric complications (diabetes, hypertension, preterm birth, anemia and cholestasis), gestational age at delivery, birth weight, Apgar scores, birth defects, admissions to ICU, perinatal mortality or puerperal problems [69]. Even though this possibility of cryopreservation extends a woman’s fertile years, it is necessary to consider that, for women who have the option of procreating earlier, using POC as a “reproductive insurance” may be risky, since nowadays it remains far from a 100% guarantee of having a successful pregnancy in the future, although in the next few years, the technical advancements might further improve the rate of successful live births.

### 5.2. Oocyte and Pregnant Women Age as Determinants of ICSI Performance

Considering data from IARTR executive summaries, we can infer that a woman who undergoes IVF/ICSI at the age of 38 using her fresh oocytes has a lower success rate than she would have using her previously frozen oocytes when she was younger or a younger donor’s frozen oocytes. Italian law does not indicate specific ages at which freezing should be carried out, thus leaving room for discretion for both the patients and the medical centers providing the procedures.

Table 2 and Table 3 report age-sensitive data for thawing techniques for the 2014–2018 and 2015–2018 periods, respectively. In Table 2, there are reported percentages of pregnancies according to oocyte age, focusing on “% pregnancies over transfers”. One can immediately observe that younger oocytes perform better than older oocytes.

Moving onto Table 3, which reports the percentage of pregnancy for fresh oocyte techniques by age of the patient we also see that “% of pregnancy over transfers” decidedly declines with age of the woman.

Considering these results, the ideal procedure would be carried out using young oocytes and an ideally young carrier. Nevertheless, oocyte donation is usually the last resort after all other options have failed, and this is the reason for the oocyte age most commonly used.

**Figure 5:**
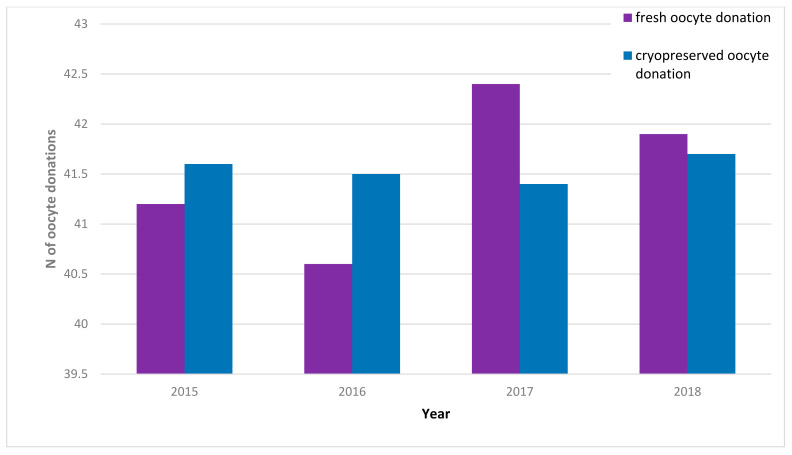
Average age of oocyte-receiving patient, 2015–2018. Italian data extracted from IARTR. The Italian Assisted Reproductive Technology Register. Executive Summary for 2018. Available online at https://www.iss.it/documents/20126/0/EXECUTIVE+SUMMARY+of+ART+in+ITALY+-+Activity+2018_.pdf/06d0b225-ac13-9be0-c20c-c94dfb6e3547?t=1617357530502 (accessed on 28 November 2021).

However, this is not what happens in practice. Figure 5 shows the average age of oocyte-receiving patients for the 2015–2018 period, where we can clearly see that for both fresh oocytes and thawed oocyte procedures the age of the mother is quite advanced. This in combination with the lack of regulation on and information about POC possibilities in Italy undoubtedly leads to procedures with characteristics opposite to the more desirable features described above; that is, the higher age of thawed oocytes and higher age of pregnant mothers after ART procedures.

**Figure 6:**
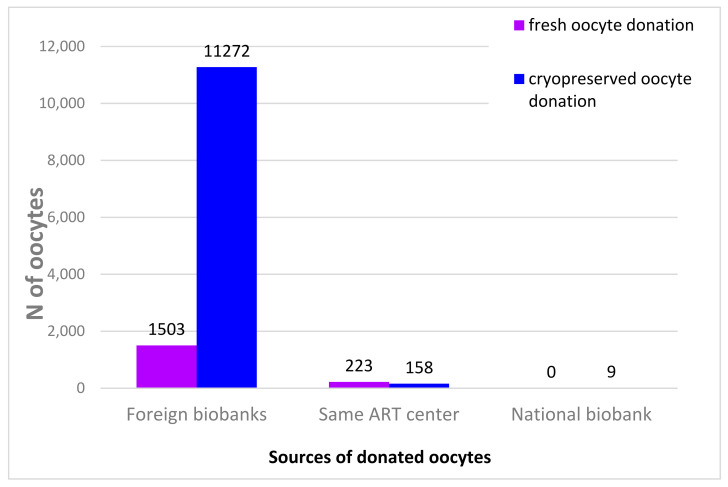
Sourcing of donated oocytes, 2015–2018. Italian data extracted from IARTR. The Italian Assisted Reproductive Technology Register. Executive Summary for 2018. Available online at https://www.iss.it/documents/20126/0/EXECUTIVE+SUMMARY+of+ART+in+ITALY+-+Activity+2018_.pdf/06d0b225-ac13-9be0-c20c-c94dfb6e3547?t=1617357530502 (accessed on 28 November 2021).

The possibility of having access to a biobank of cryopreserved oocytes donated from young women could be a highly useful alternative to using one’s fresh but older oocytes in IVF treatment. The existence of a cryobank would guarantee many advantages: each donor could donate oocytes to more than one woman; there would be no long waiting period and no need for synchronizing of the menstrual cycle between the donor and the recipient, since cryopreserved gametes would be available immediately once the endometrial preparation for the recipient is finished. Furthermore, there would be proper quarantine measurement for oocytes, improving the safety of fertility treatments for women using donated oocytes, since it would be possible to screen for potential transmittable diseases [70]. Overall, the storage of oocytes for donation seems to permit better management of the donation, with pregnancy rates comparable to fresh donor oocyte usage [36,71]. Donor availability is an ongoing issue for clinical centers; in fact, the overwhelming majority (93% in the period 2015–2018, IARTR data) of treatment cycles in Italy involving donated oocytes use oocytes from foreign biobanks, as shown in Figure 6. This is an issue not only because of higher costs in obtaining a donated gamete but also because of possible complications arising due to transport and a lack of control over supply quality.

From this perspective, a desirable policy to implement would be to encourage women who undergo elective egg freezing or standard IVF/ICSI treatments to donate supernumerary oocytes to a biobank as for other medical treatments [72], therefore making oocyte donation safer and more efficient and allowing other women who may struggle with autologous oocyte access to treatments. Encouraging donation would improve the supply of national oocytes while avoiding the possible legal and ethical complications of alternatives such as the creation of a formal or informal market for such services and would also hopefully discourage reproductive tourism for good-quality oocytes.

An interesting aspect of the discussion concerning the sale of oocytes is the hypothesis of the birth of a market for female gametes. In fact, the conservation of oocytes would not only be a benefit for those who submit to it but could also help women who need egg donors. A functional egg donation system could lead to the creation or implementation of oocyte biobanks, similar to those that already exist for sperm donors [73]. Even if the reasons that drive women to donate are purely altruistic, finding forms of compensation, even economic ones, could be a way to encourage women to donate eggs to an oocyte biobank [38]. From an economic point of view, the compensation would work in a similar way to male-gamete donation banks, for which men are paid. However, there is the danger that the compensation will become the only reason that pushes women in financial difficulties to donate, so compensation could be provided as a benefit. For example, with a donation of part of the collected oocytes, a biobank could make the maintenance of the donor’s gametes free of charge.

To minimize the possible psychosocial harm resulting from the procedure, it may be useful to limit the number of donation cycles women can undergo. Moreover, it is also important to check the emotional impact of donation on a young woman who has never had children. In fact, it could cause trauma and great psychological stress for a woman to discover that she actually has had many children born from the donation of her oocytes. Therefore, it is essential to protect both the physical and the psychological health of women, in the long term as well as from the moment of their donation. This could be achieved by clarifying the motivations of the donor and offering psychological consultation before and after donation [74].

Finally, even though the pregnancy rate does not appear to be significantly conditioned by the age of the recipient [75,76], it should still be considered that, even when using young oocytes, advanced maternal age is always problematic both for the mother and the baby. There are higher risks of pre-eclampsia, hypertension and gestational diabetes, and there is also a decline in the efficiency of maternal–fetal nutrition with respect to age, and this is associated with gestational complications, premature delivery and low birth weight with long-term effects on the child’s health [77,78,79]. Therefore, the best course of action in the context of ART seems to encourage the use of young and healthy cryopreserved oocytes (autologous or donated), preferably before the age of 40, while encouraging donation of oocytes whenever possible.

## 6. Conclusions

The undeniable trend of increasing maternal age when bearing a first child calls for the recognition of how sought after social freezing has become. Defining POC as a medical treatment for age-related fertility preservation and considering its steps as end-points to review its current safety and efficiency in Italy, it appears that the best course of action in the context of ART would be the use of young and healthy cryopreserved oocytes (autologous or donated), preferably before the age of 40, while encouraging donation of oocytes whenever possible. Italy’s dependence on foreign biobanks for donated oocytes calls for the institution of a national biobank and further regulation of gamete donation. For this reason, it would be useful to encourage the acceptance of Planned Oocyte Cryopreservation to allow greater availability of healthy, younger oocytes.

Among the possible policy proposals, considering the above-mentioned difficulties related to the coordination of POC with the current Italian legislation, a possible solution would be the introduction of a regulation specifically focused on so-called “social egg freezing”, followed by the establishment of oocyte biobanks in Italy, which would reduce the current dependence on foreign biobanks by creating a supply to match the clinical demand within the country.

Moreover, benefits could be accrued from the introduction of the correct incentives to promote gamete donation from women who undergo fertility treatments and who might want to preserve their own oocytes within the framework of future infertility prevention. Fertility preservation enacted in such a way would not constitute insurance against infertility, although the efficiency and effectiveness of techniques is ever-increasing due to recent technical progress in the field.

## Figures and Tables

**Table 1:** Birth performance indicators of fresh v. cryopreserved oocytes, 2015–2018. FOD: Fresh oocyte donation, COD: Cryopreserved Oocyte Donation; n.a.: data not available. Italian data extracted from IARTR. The Italian Assisted Reproductive Technology Register. Executive Summary for 2018. Available online at https://www.iss.it/documents/20126/0/EXECUTIVE+SUMMARY+of+ART+in+ITALY+-+Activity+2018_.pdf/06d0b225-ac13-9be0-c20c-c94dfb6e3547?t=1617357530502 (accessed on 28 November 2021).

	2015		2016		2017		2018	
	FOD	COD	FOD	COD	FOD	COD	FOD	COD
**No. of pregnancies**	40	341	49	833	15	1018	524	1430
**No. of live births**	35	246	14	633	8	740	393	1073
**No. of stillborn**	0	3	0	5	0	2	2	9
**No. of neonatal deaths**	n.a.	n.a.	n.a.	n.a.	0	6	4	4
**No. of malformed births**	0	3	0	5	0	4	3	6

**Table 2:** Percentages of pregnancies by age of oocyte, 2014-2018. PFT: pregnancies for thawing; POT: pregnancies over transfers; n.a.: data not available. Italian data extracted from IARTR. The Italian Assisted Reproductive Technology Register. Executive Summary for 2018. Available online at https://www.iss.it/documents/20126/0/EXECUTIVE+SUMMARY+of+ART+in+ITALY+-+Activity+2018_.pdf/06d0b225-ac13-9be0-c20c-c94dfb6e3547?t=1617357530502 (accessed on 28 November 2021).

	2014		2015		2016		2017		2018	
	% PFT	% POT	% PFT	% POT	% PFT	% POT	% PFT	% POT	% PFT	% POT
≤34 years old	19.3	n.a.	19.9	n.a.	17.5	21.8	19.5	23.1	21.9	26.8
35–39 years old	16.4	n.a.	14.4	n.a.	16.5	19.9	17.6	21.7	16.3	20.1
40–42 years old	12.6	n.a.	13.6	n.a.	14.1	18	12.3	15.3	11.3	15.8
≥43 years old	7.5	n.a.	16.3	n.a.	12.2	13.6	5.6	7	5.1	7.9
**Total**	**16.7**	**n.a.**	**16.6**	**n.a.**	**16.3**	**20.1**	**16.9**	**20.5**	**16.9**	**21.5**

**Table 3:** Percentages of pregnancy by age of receiving patient, 2015–2018. POSC: pregnancy over started cycles; POW: pregnancy over withdrawals; POT: pregnancies over transfers; n.a.: data not available. Italian data extracted from IARTR. The Italian Assisted Reproductive Technology Register. Executive Summary for 2018. Available online at https://www.iss.it/documents/20126/0/EXECUTIVE+SUMMARY+of+ART+in+ITALY+-+Activity+2018_.pdf/06d0b225-ac13-9be0-c20c-c94dfb6e3547?t=1617357530502 (accessed on 28 November 2021).

	2015			2016			2017			2018		
	% POSC	% POW	% POT	% POSC	% POW	% POT	% POSC	% POW	% POT	% POSC	% POW	% POT
≤34 years old	25.5	27.3	n.a.	23.6	25.3	34.7	23.5	25.2	36.1	21.7	23.1	36.6
35–39 years old	19.6	21.2	n.a.	19.8	21.5	28.7	19.4	21.1	29.8	17.9	19.4	29.6
40–42 years old	11.1	12.5	n.a.	10.6	12.1	16.6	11.4	12.8	18.4	10.3	11.7	18
≥43 years old	6.2	7.3	n.a.	4.4	5.3	8	5.6	6.8	10.8	4.9	5.9	9.8
**Total**	**17.8**	**19.6**	**n.a.**	**16.9**	**18.7**	**25.6**	**17.2**	**19**	**27.4**	**15.8**	**17.4**	**27**
